# Altered functional brain network patterns in patients with migraine without aura after transcutaneous auricular vagus nerve stimulation

**DOI:** 10.1038/s41598-023-36437-1

**Published:** 2023-06-13

**Authors:** Yuyang Rao, Wenting Liu, Yunpeng Zhu, Qiwen Lin, Changyi Kuang, Huiyuan Huang, Bingqing Jiao, Lijun Ma, Jiabao Lin

**Affiliations:** 1 Department of Psychology, School of Public Health and Management, Guangzhou University of Chinese Medicine, No.232, Huandong Road, University Town, Guangzhou, 510006 China; 2 Institut des Sciences Cognitives Marc Jeannerod, CNRS UMR 5229, Université Claude Bernard Lyon 1, Lyon, France

**Keywords:** Migraine, Sensory processing

## Abstract

Transcutaneous auricular vagus nerve stimulation (taVNS) shows excellent effects on relieving clinical symptoms in migraine patients. Nevertheless, the neurological mechanisms of taVNS for migraineurs remain unclear. In recent years, voxel-wise degree centrality (DC) and functional connectivity (FC) methods were extensively utilized for exploring alterations in patterns of FC in the resting-state brain. In the present study, thirty-five migraine patients without aura and thirty-eight healthy controls (HCs) were recruited for magnetic resonance imaging scans. Firstly, this study used voxel-wise DC analysis to explore brain regions where abnormalities were present in migraine patients. Secondly, for elucidating neurological mechanisms underlying taVNS in migraine, seed-based resting-state functional connectivity analysis was employed to the taVNS treatment group. Finally, correlation analysis was performed to explore the relationship between alterations in neurological mechanisms and clinical symptoms. Our findings indicated that migraineurs have lower DC values in the inferior temporal gyrus (ITG) and paracentral lobule than in healthy controls (HCs). In addition, migraineurs have higher DC values in the cerebellar lobule VIII and the fusiform gyrus than HCs. Moreover, after taVNS treatment (post-taVNS), patients displayed increased FC between the ITG with the inferior parietal lobule (IPL), orbitofrontal gyrus, angular gyrus, and posterior cingulate gyrus than before taVNS treatment (pre-taVNS). Besides, the post-taVNS patients showed decreased FC between the cerebellar lobule VIII with the supplementary motor area and postcentral gyrus compared with the pre-taVNS patients. The changed FC of ITG-IPL was significantly related to changes in headache intensity. Our study suggested that migraine patients without aura have altered brain connectivity patterns in several hub regions involving multisensory integration, pain perception, and cognitive function. More importantly, taVNS modulated the default mode network and the vestibular cortical network related to the dysfunctions in migraineurs. This paper provides a new perspective on the potential neurological mechanisms and therapeutic targets of taVNS for treating migraine.

## Introduction

Migraine is defined as a severe throbbing headache accompanied by sensitivity to light and sound, decreased executive ability, memory and attention, which is persistent and has severe negative impacts on sufferers' life (e.g., marital breakdown, financial strain, health decline, etc.)^1–6^. According to a 2016 study on the burden of disease, migraine has become the second leading cause of disability^7^.

Recently, magnetic resonance imaging (MRI) techniques have been employed in several migraine investigations^8–11^. The results revealed that migraineurs exhibited abnormalities in brain areas related to pain, including pain perception areas (e.g., cerebellum and brainstem) and nociceptive inhibition regions (e.g., periaqueductal gray)^8–11^. Meanwhile, migraineurs exhibited abnormalities in sensory-related areas, such as the fusiform gyrus (FFG) and insula^12–15^. Furthermore, other researches also reported unnormal brain regions relating to executive ability and attention in migraineurs, such as the paracentral lobule (PCL) and orbitofrontal gyrus (OFG)^16–19^. However, most previous researches only examined simple activation of the brain in migraine patients or performed regions of interest (ROIs) studies based on prior experience, which do not accurately reflect patterns for functional connectivity (FC) across the whole brain of migraine sufferers.

Voxel-wise degree centrality (DC) treats each voxel as an independent network node and calculates the time series correlation of each node with other voxels in the whole brain^20^. It has high retest reliability^20^. Voxels with higher DC values imply that they are located in the hub of the whole-brain network^20,21^. Thus, voxel-wise DC was used to construct brain functional connectomes and identify aberrant brain networks in certain diseases^22–24^. Specifically, DC of the cerebellum and parahippocampal gyrus was raised in elderly depressed patients compared to healthy controls (HCs); DC in the temporal lobe were found to be higher in Parkinson's patients than in HCs; brain network centrality was altered in the cerebellum and OFG of early blinded adolescents compared to normal controls^22,24,25^. Meanwhile, several studies have found that voxel-wise DC could reveal the differences in the brain networks between different subtypes of diseases, e.g., the late-onset and early-onset depression, Parkinson's syndrome in the presence or absence of cognitive impairment, schizophrenia responders and nonresponders^22,24,26^. Therefore, voxel-wise DC analysis may provide an objective and comprehensive approach to uncover specific brain FC patterns in migraine patients. Furthermore, it paves the way for further exploration of the brain mechanisms underlying transcutaneous auricular vagus nerve stimulation (taVNS) modulation of migraine patients.

Vagus nerve stimulation (VNS) has demonstrated promising potential in treating migraine^27–29^. Migraine is a central neurological disease^30–34^. The vagus nerve participates in modulating a variety of bodily functions, including inflammation, mood, somatic response and pain^35–40^. Previous studies have demonstrated evidence of the effectiveness of VNS for treating central nervous system disorders, like epilepsy, ischemic stroke, depression, etc.^41–44^. Pieces of evidence have also supported the good potential of VNS in pain relief^45,46^. Notably, several studies have shown that VNS (both invasive and non-invasive) can be effective in relieving clinical symptoms in migraineurs^29,47–49^. A randomized controlled trial revealed that migraineurs in the real taVNS had reduced headache duration, headache attacks, and headache intensity compared to migraineurs in the sham taVNS^47^. Another study reported that chronic migraineurs had significantly decreased headache duration after 1 Hz taVNS^29^. A study showed that over 40% of episodic patients reported significant pain relief after cervical vagus nerve stimulation treatment^50^. These studies show that vagus nerve stimulation is a well-established and effective technique for treating migraine. However, the mechanisms by which vagus nerve stimulation modulates brain networks in migraineurs remain unclear.

TaVNS, developed from VNS, is painless, non-invasive, portable and easy to operate^51^. Sclocco and colleagues (2020) showed that 100 Hz taVNS evoked the strongest brainstem response in healthy participants compared to other stimulation frequencies (2 Hz, 10 Hz, 25 Hz)^52^. Another study found that 8 Hz taVNS immediately reduced activation in limbic system and increased activation in insula, precentral gyrus and thalamus in healthy participants^53^. An fMRI study found that taVNS at 4/20 Hz reduced regional coherence in the frontal cortex of depressed patients^54^. In addition, Garcia et al. found that 30 Hz exhalatory-gated taVNS enhanced the connectivity of the nucleus tractus solitarii with the anterior insula and anterior middle cingulate cortex in migraineurs^55^. Compared to 20 Hz taVNS, 1 Hz taVNS reduced the functional connectivity of the locus coeruleus between the insula and anterior cingulate gyrus in migraineurs and more greatly enhanced the functional connectivity of the periaqueductal gray with the insula and anterior cingulate gyrus in migraine patients^56,57^. The above studies shows that the brain networks in migraineurs are modulated differently by selecting different frequencies of taVNS.

However, Straube et al. found that migraineurs receiving 1 Hz taVNS had significantly fewer headache days per 4 weeks than migraineurs who received 25 Hz taVNS^29^. Cao et al. showed that 1 Hz taVNS modulated the functional connectivity of pain-related brain regions in migraineurs compared to 20 Hz taVNS^56^. Meanwhile, Zhang et al. confirmed that 1 Hz taVNS was effective in relieving clinical symptoms in migraineurs^47^. These studies have highlighted the potential of 1 Hz taVNS in the treatment of migraine. However, the underlying neural mechanism by which 1 Hz taVNS modulates migraineurs is unclear.

Recently, several researches have examined the brain mechanisms regulated by 1 Hz taVNS in migraineurs^28,47,58^. An investigation of the regions of interest discovered that significantly increasing FC of the locus coeruleus and the secondary somatosensory cortex were correlated to the frequent headache onset in migraine patients after taVNS treatment (post-taVNS) than in migraine patients before taVNS treatment (pre-taVNS)^28^. In addition, another study with FC analysis using the bilateral amygdala as the ROIs showed that the real taVNS modulated FC between the amygdala and the pain network than in the sham taVNS^58^. A recent investigation revealed that taVNS could modulate the FC between thalamus subregions and the postcentral gyrus (PoCG) in migraineurs, and the FC was strongly related to a reduction in migraine attack days^47^. Notably, most studies mentioned above have used the pain-related brain regions as the ROIs to uncover the neural mechanisms of taVNS modulation. However, the modulatory effect of 1 Hz taVNS on the whole-brain functional connectivity pattern at the voxel level for migraine patients has not been considered.

In this paper, we examined the brain network features of patients with migraine without aura (MwoA) at the voxel level and the modulatory effect of 1 Hz taVNS on the brain network in migraineurs. Firstly, we employed voxel-wise DC analysis to investigate specific functional brain networks in patients compared to HCs. Secondly, to further explore the underlying mechanisms by which taVNS affects patients, the current study compared the resting-state FC in the pre- and post-taVNS patients depending on the abnormal findings of voxel-wise DC. Finally, to explore the relationship between alterations in neurological function and efficacy, we performed the correlation analysis for changes in FC and changes in clinical assessment after treatment. Based on previous researches^9,10,13,14^, we hypothesized that an abnormal brain connectivity pattern might be displayed for MwoA patients in some pivotal regions, which are involved in multisensory information integration, nociception, and cognitive function. Finally, taVNS could alleviate the migraine symptoms and modulate the FC in these brain areas.

## Participants and methods

### Participants

This paper included thirty-eight healthy controls and thirty-five episodic MwoA patients. These episodic MwoA patients were screened by experienced neurologists. The diagnostic criteria were used the beta version of the International Classification of Headache, 3rd edition^59^. Patients were required to comply with the following standards: (a) the usual hand is the right hand; (b) age is over 18 years old; (c) symptoms of migraine have lasted more than 6 months; (d) headache attacks at least twice a month (validated by self-reports from migraineurs prior to the study); (e) patients were not taking any vasoactive or psychoactive drugs during three months before the experiment. In the meanwhile, the patients' criteria for exclusion analysis were as follows: (a) other diseases causing headaches; (b) migraine episodes within two days before the MRI scan or while the scan is in progress; (c) fetation or breastfeeding period; (d) presence of additional long-term pain conditions; (e) head shape deformity or intracranially occurring lesions; (f) contraindications to MRI; (g) Self-Rating Anxiety Scale or the Self-Rating Depression Scale scores over 50.

Thirty-eight HCs recruited through advertising. The standards for inclusion in the analysis were as follows: (a) the dominant hand is the right hand; (b) the age is over 18 years old. The standards for exclusion are as follows: (a) presence of one or more primary illnesses; (b) history of alcohol abuse or family history in hereditary mental illness; (c) contraindications to MRI; (d) pregnancy or lactation; (e) history of any vasoactive or psychotropic drugs; (f) any cranial deformities or intracranial lesions.

This research strictly followed the Declaration of Helsinki. Informed consent was obtained from all participants included in the study. The Institutional Review Board of the Second Affiliated Hospital of Guangzhou University of Chinese Medicine approved this research (Z2016-079-01).

### Treatment procedure

The total duration of this study was 8 weeks. We observed the clinical presentation of the migraineurs during the first 4 weeks (baseline period) and instructed them to keep a headache diary. The content of diaries was as follows: headache duration, headache attacks, headache intensity (measured with Visual Analog Scale), quality of life for migraine (measured with Migraine Specific Quality-of-Life Questionnaire), depression status (measured with Self-Rating Depression Scale), and anxiety status (measured with Self-Rating Anxiety Scale). The patients were given the taVNS intervention and instructed to keep the diaries for the last four weeks (treatment period). Experienced acupuncturists treated patients with taVNS. The site of taVNS treatment was located in the left cymba concha, which has been shown to be a densely distributed area of the superficial vagus nerve^60,61^. The Huatuo brand electronic acupuncture treatment instrument (SDZII) was used in this study and two adjacent sites in the left cymba concha receive the taVNS stimulation (Supplementary Fig. S1). Each patient received a 4-week treatment period which included 12 sessions, with 30 min per session. Based on previous taVNS studies, patients were treated with therapy intensity of 0.2 ms and frequency setting of 1 Hz^28,47,56^. The intensity level of somatosensory stimulus was slowly adjusted to the strongest painless stimulus the patient could receive; HCs did not do any treatment.

### Resting-state functional MRI data acquisition

Patients underwent functional and structural MRI scans before and after treatment. The time window between the MRI scan and treatment visit is one day, i.e. the pre-treatment MRI scan was completed first, and then the first treatment visit was performed on the next day; at the end of treatment, the last treatment visit was performed first, and then the post-treatment MRI scan was completed on the next day. The experiment for migraineurs is shown in Supplementary Fig. S2. The HCs underwent only one scan. The same MRI scanner (Siemens MAGNETOM Verio 3.0 T, Erlangen, Germany) was used for all functional and structural MRI image scans. The scanner used a 24-channel phased-array head coil. The following parameters were employed for functional magnetic resonance imaging scans: repetition time (TR) = 2000 ms, echo time (TE) = 30 ms, field of view (FOV) = 224 mm × 224 mm, matrix = 64 × 64, flip angle = 90°, slice thickness = 3.5 mm, interslice gap = 0.7 mm, 31 axial slices paralleled and 240 time points. The detailed scanning parameters of the T1-weighted high-resolution structural images are as follows: TR = 1900 ms, TE = 2.27 ms, flip angle = 9°, FOV = 256 mm × 256 mm, matrix = 256 × 256, and slice thickness = 1.0 mm. All participants have been asked to keep clear and keep their minds off specific things. We used pillows to immobilize the patients' heads and reduce head movement. Noise generated by the MRI instrument is decreased using ear plugs. Finally, participants have been instructed to maintain their eyes shut after the start of the MRI scan.

### Data preprocessing

The preprocessing of the functional data was executed by the DPABI (V5.1) package using MATLAB^62^. The steps of preprocessing include removing the first ten time points, slice timing correction, head motion correction, normalization of the native space to Montreal Neurological Institute (MNI) space with a final size of 3 × 3 × 3 mm^3^, regression of signals from white matter, cerebrospinal fluid, and 24 head movement parameters, linear trend removal in time series from each voxel, and bandpass filtering (0.01–0.1 Hz). Notably, in the voxel-wise DC analysis, spatial smoothing using the 4-mm full width at a half-maximum Gaussian Kernel was performed after the DC calculation. Specifically, spatial smoothing with the same criteria used in DC was performed after the bandpass in the seed-based analysis.

### Voxel-wise DC analysis for MwoA patients and HCs

We used the DPABI package to perform voxel-wise DC calculations by taking each voxel as a node. Specifically, the time course of each voxel was extracted first. Subsequently, we calculated each voxel’s Pearson correlation coefficient (*r*) of the time course between each voxel and all the other voxels. Finally, the threshold of the obtained Pearson correlation coefficient matrix was set to* r* > 0.25^8,24,25,63^. In the present research, we used binary DC values^24,64^. To improve the normalization of the data, this research transformed the correlation coefficients to *z*-scores using Fisher's *r*-to-*z* transformation.

### Seed-based FC analysis for patients before treatment and after treatment

We used the DPABI package to perform FC analysis. Here, this section analyzed the modulatory effects on brain function for patients by taVNS. Firstly, the brain regions where the clusters with increased or decreased DC values were displayed in migraineurs compared to HCs were defined as ROIs. Secondly, to obtain functional connectivity maps for the pre-taVNS and post-taVNS patients, this research calculated the correlation of time series of functional connectivity between each ROI and other voxels of the brain for the patients. Notably, we employed Fisher's *r*-to-*z* transformation to increase the normalization of the correlation coefficient.

### Statistical analysis

#### Demographic and clinical assessment information

This research tested differences in demographics and clinical assessments using SPSS 26.0. Normality tests were first performed on continuous data. The Mann–Whitney U-test, Wilcoxon signed-rank test, and paired samples *t*-test were performed on the demographic data and clinical assessment data, respectively. The chi-square test was adopted to examine differences between genders.

#### Degree centrality, functional connectivity

The DPABI toolbox was adopted to analyze the obtained DC and FC between groups. Independent samples *t*-test was performed to analyze the alteration of DC values in the pre-taVNS patients and the HCs groups with age, gender, and head motions as covariates. These comparisons were used to find brain areas that differed in the patients and the HCs. Here, the gaussian random field (GRF) correction was employed for correcting multiple comparisons (voxel *p* < 0.005, cluster *p* < 0.05)^8,65,66^. In the taVNS groups, we employed a paired samples *t*-test for analyzing differences in brain FC maps in migraineurs after the treatment, with head motions as a covariate. The results have also used the GRF correction (voxel *p* < 0.005, cluster *p* < 0.05). Furthermore, for measuring the correlation between the alterations in clinical indicators (headache duration, headache attacks, headache intensity, anxiety status, depression status, and quality of life) and the alterations in brain functional connectivity in the post-taVNS patients, we employed the Pearson correlation analysis in this study.

### Institutional review board statement

The Institutional Review Board of the Second Affiliated Hospital of Guangzhou University of Chinese Medicine approved this research.

### Informed consent statement

This research strictly followed the Declaration of Helsinki, and all participants mentioned above have informed and consented.

## Results

### Demographic and clinical features in migraine patients and healthy controls

The results of population statistics and clinical data are presented in Table 1. Migraineurs have a range of 1–10 headache attacks during the baseline period, and have a range of 0–10 headache attacks during the treatment period. Gender was analyzed as a categorical variable using the chi-square test, which revealed no statistical difference in gender between patients and healthy controls. Age, headache duration, headache attacks, headache intensity, depression status, and quality of life were non-normality data after the normality tests, and anxiety status were normality data. The Mann–Whitney U-test indicated that there was no statistically significant difference in age between the migraineurs and the healthy controls. The Wilcoxon signed-rank test revealed that headache duration, headache attacks, headache intensity, depressed status, and quality of life were significantly improved in the post-taVNS patients compared to the pre-taVNS patients. Paired samples *t*-test suggested that patients had a significantly lower anxiety status after treatment.

**Table 1:** Demographic and clinical characteristics. The *p*-values for gender were obtained using chi-square tests for patients with migraines without aura group and the healthy controls. The *p*-values for age were obtained using Mann–Whitney U Tests for the MwoA patients and HCs. The *p*-values for the anxiety status were obtained using paired samples *t*-test for the pre- and post-taVNS patients. The *p*-values for the headache duration, headache attacks, headache intensity, depression status, and quality of life were obtained using Wilcoxon signed rank tests for the pre- and post-taVNS patients. *SD* standard deviation, *MwoA* migraine without aura, *HCs* healthy controls, *taVNS* transcutaneous auricular vagus nerve stimulation, *post-taVNS* after taVNS treatment, *pre-taVNS* before taVNS treatment; a, chi-square test; b, Mann–Whitney U Tests; c, Wilcoxon signed rank tests; d, paired samples *t*-test.

Characteristics	MwoA		HCs	*p* value
Gender (male/female)	10/25		14/24	0.45^a^
Age	31.97 ± 6.78		32.00 ± 11.16	0.16^b^
	pre-taVNS	post-taVNS		
Headache intensity (mean ± SD)	48.14 ± 17.13	27.71 ± 20.78		< 0.001^c^
Anxiety status (mean ± SD)	39.55 ± 5.56	36.93 ± 6.39		0.002^d^
Depression status (mean ± SD)	41.54 ± 5.23	38.32 ± 5.58		< 0.001^c^
Quality of life for migraine (mean ± SD)	60.40 ± 9.99	72.45 ± 8.89		< 0.001^c^
Headache duration (h) (mean ± SD)	53.22 ± 49.19	30.91 ± 45.71		< 0.001^c^
Headache attacks (mean ± SD)	3.89 ± 2.30	2.69 ± 2.31		0.009^c^

### Pre-taVNS vs HCs

**Figure 1:**
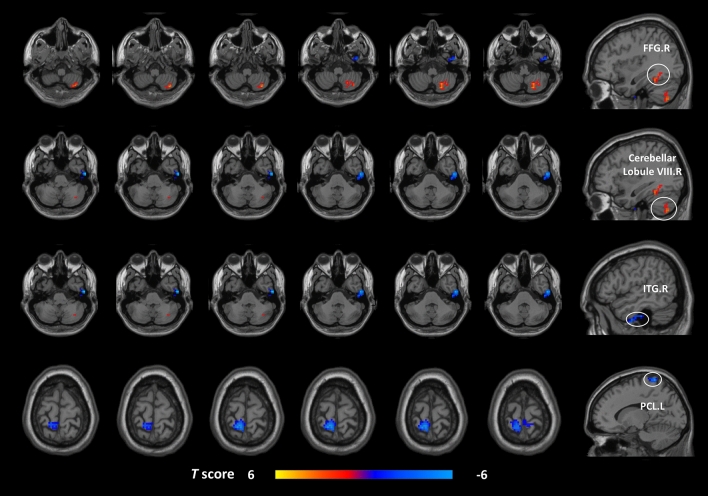
Brain regions exhibiting differences in voxel-wise DC between the MwoA patients and the HCs (GRF corrected, voxel *p* < 0.005, cluster *p* < 0.05). The color bar represents the *t*-score. Warm color represents regions with higher DC values in the patients compared to HCs; Cool color represents areas with lower DC values in the patients compared to HCs. *DC* degree centrality, *MwoA* migraine without aura, *HCs* healthy controls, *GRF* Gaussian random field, *FFG* fusiform gyrus, *ITG* inferior temporal gyrus, *PCL* paracentral lobule, *R* right, *L* left.

Voxel-wise DC analysis showed that the pre-taVNS patients had increased DC values at the right cerebellar lobule VIII and the right fusiform gyrus (FFG) compared to the HCs (Table 2, Fig. 1). In the meantime, the pre-taVNS patients had reduced DC values at the right inferior temporal gyrus (ITG) and the left paracentral lobule (PCL) than in HCs (Table 2, Fig. 1).

**Table 2:** Brain regions with different DC values between MwoA patients and HCs (GRF corrected, voxel *p* < 0.005, cluster *p* < 0.05). *DC* degree centrality, *MwoA* migraine without aura, *HCs* healthy controls, *GRF* Gaussian random field, *AAL* automated anatomical labeling, *MNI* Montreal Neurological Institute, *FFG* fusiform gyrus, *ITG* inferior temporal gyrus, *PCL* paracentral gyrus, *R* right, *L* left.

Cluster size	Brain region (AAL name)	MNI coordinates			Peak intensity
		x	y	z	
74	FFG.R	33	− 57	− 9	4.42
89	Cerebellar lobule VIII.R	21	− 63	− 48	3.78
70	ITG.R	57	− 12	− 42	− 5.01
88	PCL.L	− 9	− 45	75	− 4.22

### Pre-taVNS vs post-taVNS

**Figure 2:**
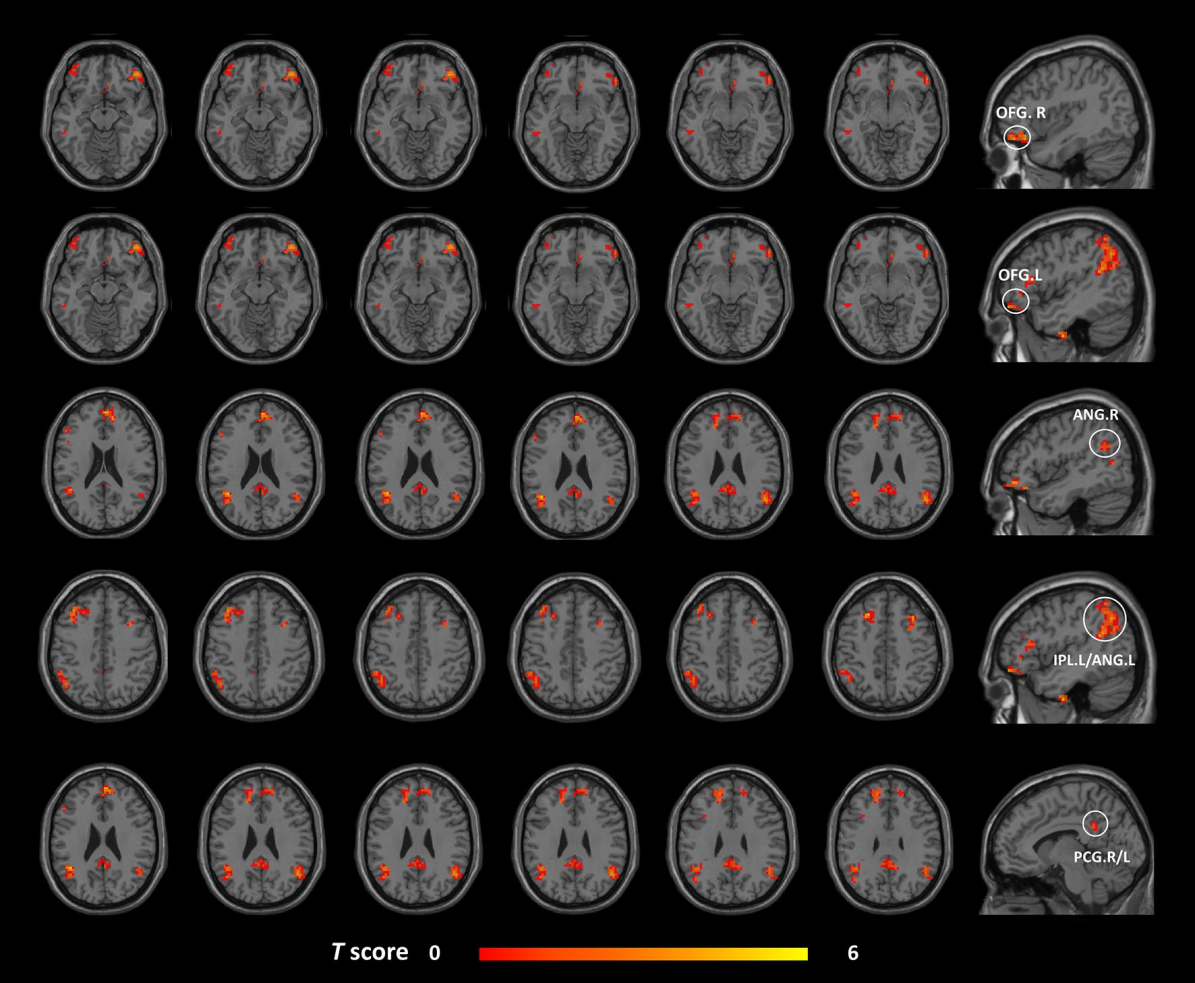
Brain regions showing increased seed-based FC between the post- and pre-taVNS patients (GRF corrected, voxel *p* < 0.005, cluster *p* < 0.05). The warm color bar represents areas where the FC of the post-taVNS patients is greater than the pre-taVNS patients; *FC* functional connectivity, *taVNS* transcutaneous auricular vagus nerve stimulation, *post-taVNS* after taVNS treatment, *pre-taVNS* before taVNS treatment, *MwoA* migraine without aura, *GRF* Gaussian random field, *OFG* orbitofrontal gyrus, *ANG* angular gyrus, *IPL* inferior parietal lobule, *PCG* posterior cingulate gyrus, *R* right, *L* left.

**Figure 3:**
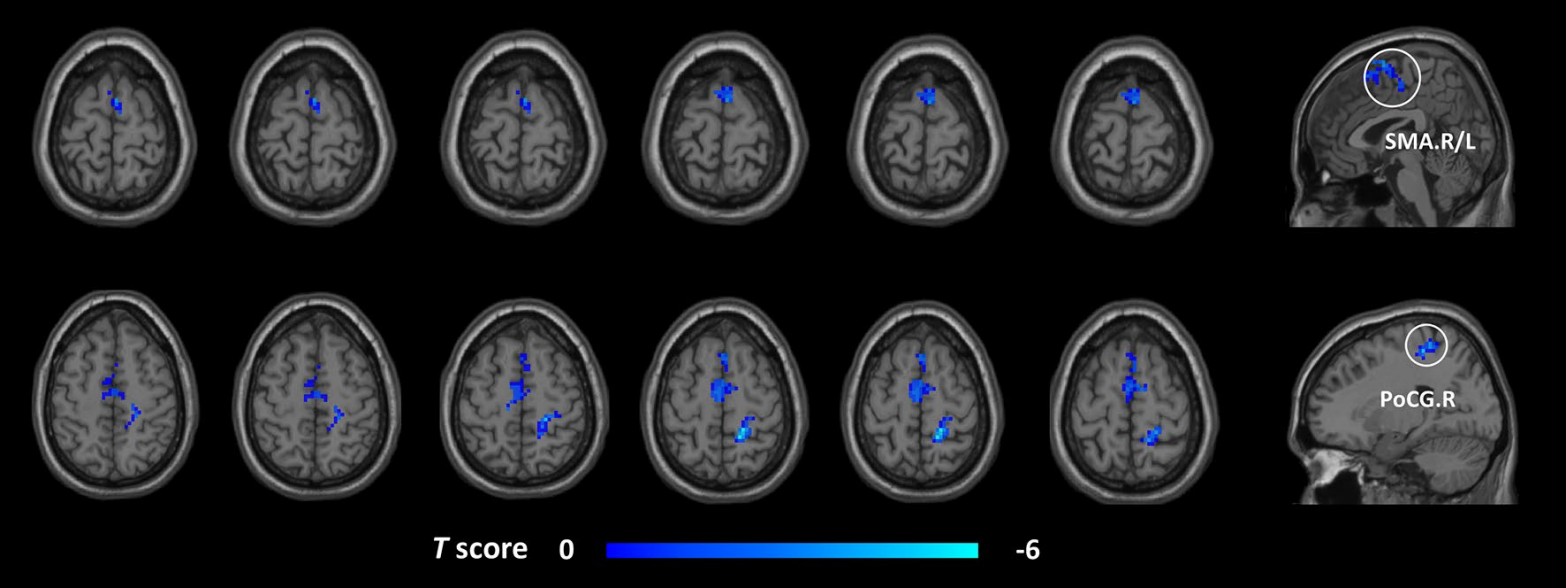
Brain regions indicating reduced seed-based FC between the post- and pre-taVNS patients (GRF corrected, voxel *p* < 0.005, cluster *p* < 0.05). The cool color bar represents areas where the FC of the post-taVNS patients is smaller than the pre-taVNS patients. *FC* functional connectivity, *taVNS* transcutaneous auricular vagus nerve stimulation, *post-taVNS* after taVNS treatment, *pre-taVNS* before taVNS treatment, *MwoA* migraine without aura, *GRF* Gaussian random field, *SMA* supplementary motor area, *PoCG* postcentral gyrus, *R* right, *L* left.

In the FC analysis, we used the right cerebellar lobule VIII, right FFG, right ITG, and left PCL as ROIs to analyze the changes in the whole-brain FC in these brain regions in the post-taVNS patients. Compared to the pre-taVNS patients, the post-taVNS patients had enhanced FC in the right ITG with the bilateral orbitofrontal gyrus (OFG), bilateral angular gyrus (ANG), left inferior parietal lobule (IPL), and bilateral posterior cingulate gyrus (PCG) (Table 3; Fig. 2). Meanwhile, compared to the pre-taVNS patients, the post-taVNS patients had reduced FC of the right cerebellar lobule VIII with the bilateral supplementary motor area (SMA) and right PoCG (Table 4; Fig. 3). The correlation analysis revealed that the changed FC of ITG-IPL was significantly and positively correlated with the changes in headache intensity.

**Table 3:** Brain regions showing altered FC with the ITG in the post-taVNS patients compared to the pre-taVNS patients (GRF corrected, voxel *p* < 0.005, cluster *p* < 0.05). *FC* functional connectivity, *ITG* inferior temporal gyrus, *taVNS* transcutaneous auricular vagus nerve stimulation, *post-taVNS* after taVNS treatment, *pre-taVNS* before taVNS treatment, *GRF* Gaussian random field, *AAL* automated anatomical labeling, *MNI* Montreal Neurological Institute, *OFG* orbitofrontal gyrus, *ANG* angular gyrus, *IPL* inferior parietal lobule, *PCG* posterior cingulate gyrus, *L* left, *R* right.

Cluster size	Brain region (AAL name)	MNI coordinates			Peak intensity
		x	y	z	
79	OFG.R	36	33	− 18	4.85
28	OFG.L	− 39	21	12	4.61
52	ANG.R	51	− 60	27	4.44
99	ANG.L /IPL.L	− 42	− 51	24	4.73
33	PCG.R/L	− 6	− 42	36	3.92

**Table 4:** Brain areas indicating changed FC with the cerebellar lobule VIII in the post-taVNS patients compared to the pre-taVNS patients (GRF corrected, voxel *p* < 0.005, cluster *p* < 0.05). *FC* functional connectivity, *taVNS* transcutaneous auricular vagus nerve stimulation, *post-taVNS* after taVNS treatment, *pre-taVNS* before taVNS treatment, *GRF* Gaussian random field, *AAL* automated anatomical labeling, *MNI* Montreal Neurological Institute, *SMA* supplementary motor area, *PoCG* postcentral gyrus, *L* left, *R* right.

Cluster size	Brain region (AAL name)	MNI coordinates			Peak intensity
		x	y	z	
200	SMA.R/L	3	3	69	− 4.10
33	PoCG.R	18	− 39	60	− 5.10

## Discussion

A study has suggested that the neural mechanisms of episodic migraine were different to chronic migraine^67^. However, our study focused on patients with episodic migraine without aura. This research first examined specific brain network patterns in episodic migraineurs without aura than in the HCs with a DC method at the voxel level. Using the areas that had alterations in DC values of the pre-taVNS patients compared to the HCs, we performed whole-brain FC analysis to test whether the taVNS could modulate the neural mechanisms underlying the patients before and after treatments. We found increased DC of FFG and cerebellar lobule VIII, and decreased DC of ITG and PCL in the pre-taVNS patients than HCs. Previous studies have shown that taVNS can modulate brainstem regions in migraine patients^55^. However, our study focused on abnormal brain networks in migraineurs compared with HCs and explored the modulation of these abnormal brain networks by taVNS. Our study showed that compared with the pre-taVNS patients, taVNS can significantly modulate the FC of these specific brain regions in migraineurs. Headache duration, headache attacks, and headache intensity were significantly reduced in patients after treatment. Finally, we discovered a correlation between the changes in FC of the ITG-IPL and the changes in headache intensity in the patients.

### Altered brain connectivity in MwoA patients than healthy controls

We found elevated DC values of the FFG, cerebellar lobule VIII, and reduced DC values of the ITG in the patients than in the HCs. It suggested that alterations in central locations of the brain network in these brain regions are associated with migraine. The FFG is an important part of higher visual function and participates in injurious/anti-injurious sensory processing^68–70^. In agreement with our study, previous researches have demonstrated that FFG is abnormally altered in migraineurs compared to HCs, including reduced cortical thickness and decreased amplitude of low-frequency fluctuation (ALFF)^13,71^. The cerebellum is a multi-modal region involved in integrated sensory information processing, pain processing, and motor control^72–74^. The finding in our paper is consistent with other earlier studies^9,13^. To be specific, a functional MRI (fMRI) study has shown that ALFF and fractional ALFF in the cerebellum were significantly reduced in migraineurs compared to HCs^13^. A hemodynamic investigation of migraine patients showed ischemic lesions in the cerebellar lobes^9^. The ITG is a multisensory information integration brain region associated with visual perception^8,75,76^. An MRI study on migraine indicated an increased FC of ITG and lingual gyrus in migraineurs than in HCs^77^. The FFG and ITG belong to the visual cortex, and they are adjacent to the anatomical location of the cerebellum^68,78^. Migraine sufferers experienced sensory hypersensitivity, including abnormal multisensory integration, both during and between headache attacks^79–81^. Visual and auditory stimuli could enhance the intensity of headaches in migraineurs^82^. Compared to non-migraineurs, migraineurs are hyper-aware of everyday sounds (car horns, bells), which could cause migraine attacks^83–85^. Therefore, altered DC values in the FFG, ITG and cerebellar lobule VIII might suggest aberrant multisensory integration and pain perception in MwoA patients.

In addition, the pre-taVNS patients had reduced DC values in the PCL compared to HCs. This finding suggested an association between altered brain connectivity in PCL and migraine. The PCL is primarily associated with memory and attentional functions^86–88^. The majority of migraineurs have exhibited abnormal cognitive functions in certain brain regions^1,2^. Compared to normal individuals, migraineurs had decreased sustained attention^89^. In the word recall experiment, migraineurs had lower memory scores than the HCs^1,2,89^. Additionally, prolonged migraine headaches might impair cognitive ability^90^. Compared to the HCs, migraineurs had lower scores on the Montreal Cognitive Scale^2,89^. Consistent with our study, a previous retrospective fMRI study found altered PCL cortex thickness in childhood migraineurs^14^. Thus, the altered brain connectivity in the PCL might be related to cognitive impairment in MwoA patients.

### Behavioral and neural modulation effects of the taVNS treatments for MwoA patients

#### Clinical symptoms

We found that taVNS significantly alleviated headache and concomitant symptoms in the post-taVNS patients. In other words, it mainly reduced headache intensity, headache attacks, and headache duration. These results aligned with previous studies^27,29,47^. Zhang et al. found a significant reduction in headache symptoms in migraineurs after taVNS treatment^47^. Other research discovered that taVNS of 1 Hz could reduce the days of the migraine attack in chronic migraineurs compared to 25 Hz^29^. Therefore, we believe that taVNS is promising for treating migraine.

### Altered FC of the ITG in the post-taVNS MwoA patients

In this study, the taVNS significantly modulated the FC of the temporal lobe in patients following treatments. The FC of the ITG with the ANG, IPL, PCG, and OFG were enhanced in the post-taVNS patients compared to the pre-taVNS patients. The ITG is connected to the ANG by the arcuate fasciculus^76^. The ITG, ANG, PCG, and IPL are included in the default mode network (DMN)^91,92^. When it comes to pain processing, the DMN is crucial^93^. In the studies of pain disorders, it has been proved that the DMN may be involved in multiple chronic pain disorders^94–97^. Similar to our study, previous researches revealed altered FC within the DMN of migraineurs^8,98^. The IPL, including the ANG, is regarded as a hub for information transmission and integration^99^. Numerous investigations have revealed that IPL is associated with a wide variety of cognitive impairments^100–102^. Meanwhile, several studies have found altered FC of the IPL in migraineurs than in healthy controls^103–105^. The PCG is also thought to be involved in transmitting and integrating information^106^. A study proved that cortical thickness of PCG in treated migraine patients was negatively associated with improvements in headache index^107^. It has been proposed that migraine affects the nervous system mainly in terms of sensory information transmission and integration; in other words, migraine may be a disorder with altered multisensory integration^108^. For example, the perception of normal touch, sound, smell and light is amplified in migraineurs^109^. Meanwhile, hyper-perception may lead to headaches or worsen headaches' intensity^83–85^. As a result, altered FC between the ITG with the ANG, PCG, and IPL in post-taVNS patients may indicate that the DMN might be involved in the modulatory effect of taVNS on MwoA patients. TaVNS might be able to modulate abnormal multisensory (light, sound, pain perception) information integration and transmission on migraineurs.

The OFG is a brain region closely related to cognitive and executive functions^110^. Migraineurs have significantly high cerebral blood flow in OFG than in HCs^111^. Most migraineurs struggle with executive function or decision-making^112,113^. Compared to the HCs, migraineurs showed executive dysfunction related to headache duration and intensity^114^. Therefore, the post-taVNS patients with enhanced FC of the ITG and OFG indicated that taVNS might regulate the connectivity in these brain regions related to multisensory information processing and executive functions in MwoA patients.

In addition, the changed FC of the ITG and IPL was significantly correlated to changes in headache intensity. We supposed that symptomatic clinical remissions in post-taVNS patients might be explained by FC changes in the ITG and IPL.

### Altered FC of the posterior cerebellar lobes in MwoA patients after taVNS treatments

Our study indicated that the cerebellum of patients after taVNS has fewer functional connectivity to SMA and PoCG. The cerebellum, SMA, and PoCG are part of the vestibular cortical network (VCN)^115^. The vestibular cortical network involves motor balance and spatial navigation^116^. Abnormal activation of the VCN is found in migraine patients compared to HCs^105^. Follow-up of migraineurs shows that they often feel persistent vertigo, affecting their quality of life^117^. Vertigo symptoms in migraine patients may persist throughout the illness^118,119^. Regarding neural projections, the SMA receives fibre projections from the cerebellum^120^. Several pain-related studies have identified abnormal alterations in the SMA^121,122^. PoCG is involved in identifying pain information^123^. Meanwhile, the PoCG is the primary somatosensory cortex that regulates the corresponding behaviors based on sensory information^124^. Moreover, the FC of the PoCG was altered in resting-state fMRI investigations of migraine^8,27,125^. Thus, taVNS might participate in modulating the intrinsic connectivity within the VCN of MwoA patients, which is a brain network associated with homeostasis. Meanwhile, taVNS may modulate the brain functional connectivity patterns associated with the pain of MwoA patients.

### Limitations

A few limitations are associated with this research. First of all, our study lacked an additional control group (migraineurs undergoing shame treatment or healthy subjects undergoing real treatment). On the one hand, our experiment was conducted in the hospital, which has a high mobility of healthy participants who are unwilling to participate in long-term experimental tasks. On the other hand, many migraineurs have feelings of anxiety or depression, they are unwilling to undertake additional experimental tasks. In addition, we have only studied the short-term effects of taVNS on migraineurs in the present study. However, some studies have also considered the long-time effect of taVNS^126,127^. For future research, it should be considered to investigate the long-term effects of taVNS in migraineurs. Finally, gender differences were not considered in our fMRI study. An epidemiological study revealed that women get migraines at considerably greater rates than males do^128^. Exploring the effects of taVNS on gender-specific migraine patients is an interesting idea that we will explore further in a subsequent study.

## Conclusion

Our research suggested that MwoA patients have altered brain connectivity patterns in several hub regions, including ITG, FFG, cerebellar lobule VIII, and PCG, which are related to multisensory integration, pain perception, and cognitive function. Meanwhile, we found that taVNS significantly modulates the specific functional brain networks in MwoA patients, such as the default mode network and the vestibular cortical network. The discoveries in this research might help provide insight into the neurological mechanics of migraine and provide some evidence to explore neural therapeutic targets of taVNS against migraine.

## Supplementary Information

Supplementary Figures.

## Data Availability

The data presented in this study are available on request from the corresponding authors.
