# Supplementary material for: Altered functional brain network patterns in patients with migraine without aura after transcutaneous auricular vagus nerve stimulation
Source: Sci Rep. 2023 Jun 13;13:9604. doi: 10.1038/s41598-023-36437-1 (PMC10264378; doi:10.1038/s41598-023-36437-1)
Supplement: Supplementary file 1 — Supplementary Figures. [file 41598_2023_36437_MOESM1_ESM.docx]

**Supplementary Information**


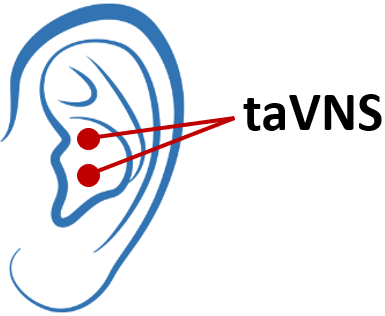


**Fig. S1. A schematic of taVNS delivered sites.**

Abbreviation: taVNS, transcutaneous auricular vagus nerve stimulation.


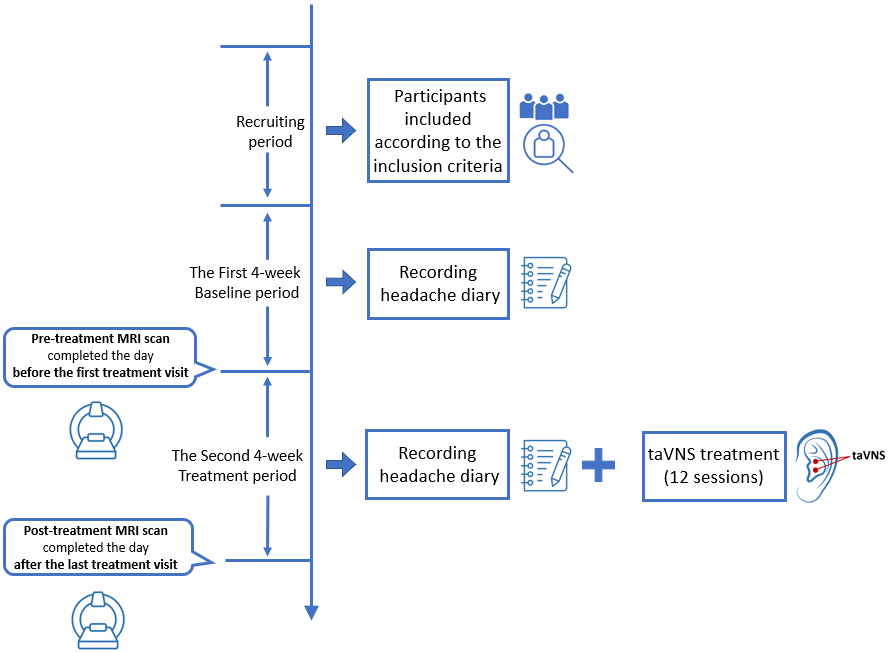


**Fig. S2. A flowchart of the experiment for migraineurs.**

Abbreviation: taVNS, transcutaneous auricular vagus nerve stimulation.
